# VirtualFlow Ants—Ultra-Large Virtual Screenings with Artificial Intelligence Driven Docking Algorithm Based on Ant Colony Optimization

**DOI:** 10.3390/ijms22115807

**Published:** 2021-05-28

**Authors:** Christoph Gorgulla, Süleyman Selim Çınaroğlu, Patrick D. Fischer, Konstantin Fackeldey, Gerhard Wagner, Haribabu Arthanari

**Affiliations:** 1 Department of Physics, Harvard University, Cambridge, MA 02138, USA; 2 Department of Biological Chemistry and Molecular Pharmacology, Harvard Medical School, Boston, MA 02115, USA; PatrickD_Fischer@dfci.harvard.edu (P.D.F.); gerhard_wagner@hms.harvard.edu (G.W.); 3 Department of Cancer Biology, Dana Farber Cancer Institute, Boston, MA 02115, USA; 4 Department of Biochemistry, University of Oxford, South Parks Road, Oxford OX1 3QU, UK; suleyman.cinaroglu@bioch.ox.ac.uk; 5 Department of Pharmacy, Pharmaceutical and Medicinal Chemistry, Saarland University, 66123 Saarbrücken, Germany; 6 Zuse Institute Berlin, 14195 Berlin, Germany; fackeldey@zib.de; 7 Institute of Mathematics, Technical University Berlin, 10623 Berlin, Germany

**Keywords:** structure based virtual screening, molecular docking, swarm intelligence, artificial intelligence, computer aided drug design, CADD, KEAP1, drug discovery

## Abstract

The docking program PLANTS, which is based on ant colony optimization (ACO) algorithm, has many advanced features for molecular docking. Among them are multiple scoring functions, the possibility to model explicit displaceable water molecules, and the inclusion of experimental constraints. Here, we add support of PLANTS to VirtualFlow (VirtualFlow Ants), which adds a valuable method for primary virtual screenings and rescoring procedures. Furthermore, we have added support of ligand libraries in the MOL2 format, as well as on the fly conversion of ligand libraries which are in the PDBQT format to the MOL2 format to endow VirtualFlow Ants with an increased flexibility regarding the ligand libraries. The on the fly conversion is carried out with Open Babel and the program SPORES. We applied VirtualFlow Ants to a test system involving KEAP1 on the Google Cloud up to 128,000 CPUs, and the observed scaling behavior is approximately linear. Furthermore, we have adjusted several central docking parameters of PLANTS (such as the speed parameter or the number of ants) and screened 10 million compounds for each of the 10 resulting docking scenarios. We analyzed their docking scores and average docking times, which are key factors in virtual screenings. The possibility of carrying out ultra-large virtual screening with PLANTS via VirtualFlow Ants opens new avenues in computational drug discovery.

## 1. Introduction

When a drug (ligand) enters the body, it typically engages a specific binding site on a chosen target protein and triggers the desired therapeutic effect. The tighter the ligand binds to the target protein, the higher its efficacy at much lower concentrations. This would require much lower concentrations of the drug, avoiding toxicity due to off target effects. Since the ligand as well as the target protein are not static in nature, but flexible and dynamic in solution, different conformations and orientations of the ligand–target protein complex are possible, and each of these conformations would result in different binding affinities (binding strengths). Hence, the process of computationally finding the optimal ligand–protein complex can roughly be divided into two steps: In the first step, all possible spatial arrangements of the ligand and the target protein are enumerated. To this end, the two structures must be brought together and arranged in various different spatial arrangements; this process is called docking. However, not all orientations and conformations of the ligand–target complex lead to the same binding affinity or binding strength. Therefore, in a second step, a score is assigned to each spatial arrangement of the ligand and the protein. The spatial constellation with the lowest score (i.e., the free energy of binding) then represents the strongest binding of the ligand and the binding site of the target, in other words: the “best” fit. Assuming that one assigns a score for each possible arrangement of the ligand with respect to the protein, one obtains a scoring function or binding affinity function, which assigns a free energy of binding to each conformation. This binding affinity function can be considered as the ground truth whose absolute minimum constitutes the best fit of the protein–ligand complex. However, unfortunately, this function is hardly computable, even for small systems. Therefore scoring functions are used which try to approximate the “true” energy binding functions.

Analogous to the binding energy function, a scoring function assigns a value to each computed orientation/conformation scenario, a score which typically correlates with the binding energy. In the same way, the absolute minimum represents the constellation with the highest binding affinity. For most docking programs, this means that the more negative the docking score, the stronger the predicted binding affinity. Analogous to the energy binding function, one is interested in the absolute minimum of the scoring function. In the last decades, different methods have been developed, which mainly differ in the way of posing (ligand flexible/protein fixed, ligand flexible/protein flexible, ...), in the choice of degrees of freedom of the scoring function and in the search method for the minimum, among them are DOCK [1], AutoDock [2], GOLD [3] or FlexX [4]), to mention a few.

In mathematical terms, the scoring function of a given target–ligand complex described by the *n* degrees of freedom $x={\left(x_{1},...,x_{n}\right)}^{T}\in R^{n}$, is an objective function $f:R^{n}\to R$. Hence, the docking problem can be expressed as a (global) optimization problem, i.e.,

$$\min_{x\in R^{n}}f\left(x\right).$$

Summing up, finding the best arrangement of the protein and the ligand is equivalent to finding the (absolute) minimum of *f*. In the following section, we will treat this problem by taking advantage of agent based methods developed in the context of swarm intelligence.

## 2. Ant Intelligence in Molecular Docking with PLANTS

In agent-based models, the global behavior of a system is employed by local interactions between agents on a micro scale. The PLANTS (Protein-Ligand ANT System) [5,6,7] is a stochastic optimization method for non-covalent protein–ligand docking algorithms, which is based on ant colony optimization (ACO) [8,9].

ACO itself is inspired by the swarm intelligence of ants: Each ant deposits a substance (pheromone) that other ants sense. If an ant has several choices, it follows with a higher probability the way with the highest pheromone concentration. If a certain trail is shorter than other trails, more ants will use it in the same time interval, which leads to a higher pheromone concentration. Contrariwise, if a trail is not used for a longer time span, the concentration of pheromone decreases (evaporation). At the end, the whole ant colony has optimized its way. Setting this into the context of the protein–ligand docking, the shortest trail can be related to the structure with the lowest binding energy.

The ACO algorithm optimizes the trail between the ant nest (=initial position of the molecule) and the food source (=target position). ACO belongs to the class of agent-based swarm models, where the behavior of a complex system is investigated by interaction between agents (ants). Originally, the ant algorithm was used for combinatorial problems like finding the shortest path in a given network. Here, we have no network, but a function and seek for its absolute minimum. Thus, in order to apply the ACO algorithm to our problem, we need a discretization for the degree of freedom and an iteration rule of the ants.

### 2.1. Discretization

Let us restate that the spatial orientation of the target–ligand complex is given by a *n* dimensional vector $x={\left(x_{1},...,x_{n}\right)}^{T}\in R^{n}$ where each degree of freedom $x_{i}\in \left[a^{i},b^{i}\right]$ is a real number. However, for applying the ACO, we can only permit finitely many values for each degree of freedom.

We therefore partition each interval $\left[a^{i},b^{i}\right]$ by a finite series

$$a^{i}=z_{i}^{0}<z_{i}^{1}<....<z_{i}^{n_{i}}=b^{i}$$

into $n_{i}$ sub intervals, i.e.,

$$\left[a^{i},b^{i}\right]=\overset{n_{i}}{\underset{\ell =1}{⋃}}\left[z_{i}^{\ell -1},z_{i}^{\ell }\right].$$

By introducing the characteristic function

$$\chi_{i,\ell }\left(x_{i}\right)=\{\begin{array}{cc} z_{i}^{\ell } & \mathrm{if}x_{i}\in \left[z_{i}^{\ell -1},z_{i}^{\ell }\right]\\ 0 & \mathrm{otherwise}. \end{array}\;\;\;\ell =1,...,n_{i}$$

we can assign to each real value $x_{i}$ an interval value $z_{i}^{\ell }$. For instance, let $x_{i}$ be the rotational degree of freedom, then the partition [0,360]=[0,1]∪[1,2]∪...∪[359,360] can be used. A value of 1.7 would be assigned to the second interval and $\chi_{i2}\left(1.7\right)=1$


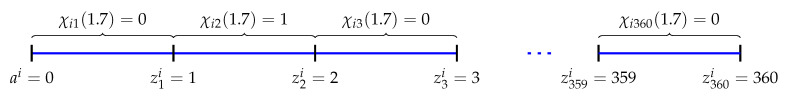


To date, we have just employed a discretization scheme for the degrees of freedom, such that the real valued degrees of freedom can be assigned to integers.

### 2.2. Iteration Rule of the Ants

The movement of the ants is represented as an iterative process. At time *t*, the ant a is in a certain position $x^{a}\left(t\right)$. Then, for each degree on freedom *i*, a local minimization by the simplex algorithm is employed, i.e.,

$$s_{i}^{a}={\mathrm{argmin}}_{x_{i}^{a}\in R^{n}}f\left(x_{i}^{a}\right),i=1,...,n$$

such that $s^{a}$ is a local minimizer of *f* in the iteration step *t*. In the original ant algorithm, the ants walk on a graph. An ant that is at time step *t* on node *i* then chooses the edge *ℓ* with probability

(1) $$p_{i\ell }\left(t\right)=\frac{\tau_{i\ell }\left(t\right)}{\sum_{k=1}^{n_{i}}\tau_{ik}\left(t\right)},$$

where $\tau_{i}^{\ell }\left(t\right)$ is the so called pheromone trail, which assigns a desirability of edge *ℓ* at time step *t*. In the context of PLANTS (1) is the probability to assign $x_{i}$ to $z_{i}^{\ell }$. With this, the pheromone trails $\tau_{i}^{\ell }$ can be updated via

$$\tau_{i\ell }\left(t+1\right)=\left(1-\rho \right)\tau_{i\ell }\left(t\right)+1_{i\ell }\Delta \tau_{i},$$

where

$$1_{i\ell }=\left\{\begin{array}{cc} 1 & \mathrm{if}s_{i}^{a}\in \left[z_{i}^{\ell -3},z_{i}^{\ell +2}\right]\mathrm{and}x_{i}\mathrm{is}\mathrm{rotational}\mathrm{dof}\\ 1 & \mathrm{if}s_{i}^{a}\in \left[z_{i}^{\ell -2},z_{i}^{\ell +1}\right]\mathrm{and}x_{i}\mathrm{is}\mathrm{not}\mathrm{rotational}\mathrm{dof}\\ 0 & \mathrm{otherwise}. \end{array}\right.$$

and

$$\Delta \tau \left(s_{i}^{a}\right)=\left\{\begin{array}{cc} \left|f(x_{i}^{a})\right| & \mathrm{if}f(x_{i}^{a})<0\\ 1 & \mathrm{if}s_{i}^{a}\in \left[z_{i}^{\ell -2},z_{i}^{\ell +1}\right]\mathrm{and}x_{i}\mathrm{is}\mathrm{not}\mathrm{rotational}\mathrm{dof}\\ 0 & \mathrm{otherwise}. \end{array}\right.$$

**Figure 1:**
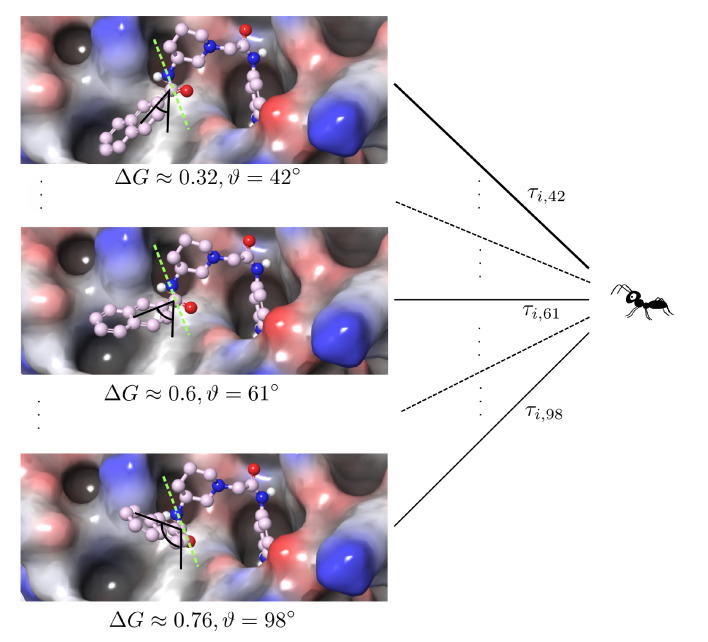
Sketch of a one the *i* th degree of freedom with $x_{i}=\vartheta \in \left[0,360\right]$. The spatial arrangement with the angle $\vartheta =42^{\circ }$ has the lowest energy (ΔG≈0.32), such that $\tau_{i,42}$ has the highest value and consequently the ant chooses the trail “42” with the highest probability.

This principle is illustrated in Figure 1.

For an upper and lower bound of the pheromone trails for each degree of freedom, we refer to [7]. In PLANTS, the candidate solutions are optimized by the simplex local search algorithm [10].

### 2.3. PLANTS Features for Molecular Docking

PLANTS provides many special features for molecular docking studies, as well as nature-inspired molecular docking algorithm. PLANTS contains three empirical scoring functions: PLP (piece-wise linear potential), PLP95, and ChemPLP [6,11,12]. Piece-wise linear potentials are used to model the steric complementarity between the protein and ligand in all of the three scoring functions. All scoring functions use potentials for van der Waals interactions and repulsive terms. An internal score of the ligand contains an empirical heavy-atom potential to avoid internal ligand clashes. Furthermore, the torsional potential from the Tripos force field [13] is calculated for rotatable bonds in the ligand. ChemPLP, in addition, contains angle-dependent hydrogen bond terms from GOLD’s ChemScore [14,15]. ChemPLP is the default scoring function in PLANTS and also in GOLD. PLANTS allows one to modify and customize many intermolecular and intramolecular terms which are used by these three scoring functions. PLANTS cannot only be used in classical dockings and virtual screenings, it also allows to rescore compounds for given protein–ligand complexes. PLANTS uses the MOL2 file format for both the receptor and the ligands, having the advantage of being one of the most commonly used chemical file formats, which is able to store atom positions, connectivity, and arbitrary meta information in a single file. This information is extremely important for the identification of rotatable bonds and functional groups in molecular docking. MOL2 is a standardized file format that can be read by other modeling programs.

Many features of the PLANTS docking program make it a powerful tool for molecular docking. With the fastest setting PLANTS can be used to economically sample vast chemical spaces. Additionally, PLANTS allows flexibility on the receptor side, i.e., on side chains of the amino acid residues in the protein. Even if no flexibility is specified, PLANTS partially applies the flexibility to the protein by optimizing the positions of hydrogen atoms. On the ligand side, PLANTS can also perform rigid-body docking, allowing for externally generated ligand conformations. It is also possible to perform rigid ligand docking with flexible protein side chains.

PLANTS has also been compared to other docking tools, among them are [16,17,18,19,20]. In [16], for instance PLANTS has been tested on the human cluster of differentiation 38 (CD38), in the ranking based on scoring power with 42 compounds PLANTS was in the upper class.

One of the challenges of high-throughput docking routines is the treatment of the effect of water and its contribution to the binding free energy. Before the ligand engages the protein, both the protein and the ligand are solvated. Upon binding, there will be changes in the solvation. It is easy to conceive that water molecules will be freed from the ligand upon binding the protein and this would result in an entropic gain. On the side of the protein, solvation is more complex. It has been shown that in the case of protein–small molecule interactions, one of the major entropic contributions to the binding free energy stems from desolvation of the small molecule upon binding, especially in case of small molecule harboring hydrophobic groups [21,22]. High-throughput docking routines traditionally use a version of an implicit water model which enables screening large libraries of compounds in reasonable time. However if the docking interface on the protein has bound water molecules with long resident times, and they could contribute significantly to the binding free. This contribution will not be appropriately captured by the implicit water mode. Sometimes, the water molecules bound to the protein might help for stabilizing the ligand–protein complex via mediating hydrogen-bonding interactions. In high-resolution crystal structures, typically with resolution better than 2.8 *Å*, we potentially observe highly ordered water molecules. Occasionally, these water molecules would aid connecting the protein to the ligand in the binding pocket [23]. PLANTS has features to include explicit water molecules during the molecular docking, which allows the water molecule to move by translation or rotation. It is also possible to fix all of the water molecule’s degrees of freedom. This capability also allows to optimize hydrogen bond parameters of the water.

PLANTS can help lead optimization by scaffold hopping via restraining the position of a ring system or a non-ring atom in molecular docking. All degrees of freedom in the scaffold can be completely neglected using this approach. So, many different substituents on the scaffold can be screened. This is a useful feature for lead optimization since all manipulations in the use of scaffold hopping in medicinal chemistry generally involve cyclic systems [24].

While it is desired to have a high-resolution crystal structure of the ligand bound to the protein, there are several instances where we are unable to co-crystallize the protein–ligand complex or instances where we do not see the density for the ligand. In some of these instances, nuclear magnetic resonance (NMR) spectroscopy can provide information that can help docking routines. In an ideal case nuclear Overhauser effect (NOE), restraints between the ligand and protein would provide distance information to obtain the co-structure. However, in many cases, we might not be able obtain these constraints due to a number of reasons, including the broadening of the ligand signals due to increase in size or exchange kinetics or the absence of hydrogen atoms on the ligand at the binding interface. In the absence of well defined NOE restraints, there are a series of ligand-detected NMR experiments like trNOE [25], saturation transfer difference (STD) [26], DIRECTION [27] and INPHARMA [28] experiments that can provide qualitative information on pharmacophore mapping. PLANTS also allows molecular docking with NMR constraints derived from ligand-detected NMR experiments [29,30]. PLANTS can use these experimental data to evaluate the poses and to identify the pose fitting the experimental constraints. This approach can be helpful in case of having highly flexible ligands since it may be very difficult to predict all degrees of freedom for these ligands. Thus, the experimental data can be a part of the docking algorithm to help finding correct binding poses of the ligands with more reliable predictions.

Using molecular interaction fingerprints (IFP) can even increase the accuracy in molecular docking using PLANTS. PLANTS has an ability to generate IFP by running PLANTS in rescore-mode with one or multiple ligands for a given protein structure. After IFP identification, more robust structure-based virtual screening can be performed using these IFP, which can be set as a constraint. Multiple IFP files can be specified during the docking. In addition to these constraints, many other constraints can be specified in molecular docking with PLANTS [31]. The docking predictions will be influenced by the constraints, including hydrogen bond constraint, shape constraint and distance constraints.

## 3. VirtualFlow Ants—Virtual Screenings Using Ant Intelligence via PLANTS

We have added support to the docking program PLANTS in our recently developed drug discovery platform VirtualFlow [32,33]. This new feature is called VirtualFlow Ants; it also adds support for a new ligand preparation routine involving the MOL2 format, to enable screening using PLANTS. We have tested the scaling behavior of VirtualFlow Ants using a protein Kelch-like ECH-associated protein 1 (KEAP1) as a model system, and studied how varying some of the key parameters in the docking program PLANTS affects the results of the virtual screenings with our test system.

### 3.1. Ligand Preparation and Chemical File Formats

**Figure 2:**
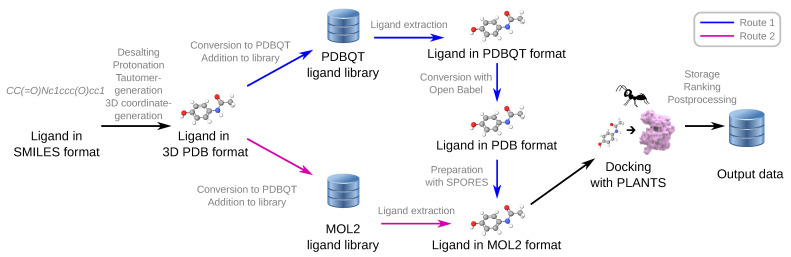
Overall ligand workflow in VirtualFlow Ants. Ligand libraries can be provided in two formats, firstly the PDBQT format and secondly the MOL2 format. New libraries in either format can be prepared with VirtualFlow for Ligand Preparation (VFLP). If the ligand library is provided in the PDBQT format (Route 1 in blue), then VirtualFlow Ants will convert and prepare the molecules on the fly into the MOL2 format (with Open Babel and SPORES) which is required by PLANTS. If the ligand library is provided in the MOL2 format, then no on the fly conversion is required (Route 2 in magenta), and the ligand can simply be extracted from the ligand library during the virtual screens.

The virtual screening module of Virtual Flow, VirtualFlow for Virtual Screening (VFVS), previously only supported ligands in the PDBQT format [34,35] because all previously supported docking programs were to use this chemical file format and some of them only supported this single file format like AutoDock Vina or QuickVina 2. PLANTS, however, requires the ligands to be in the MOL2 format. To meet this requirement, we have added two different implementations. First, we added the support of ligands in the MOL2 format to VirtualFlow Ants. The overall structure of the input ligand libraries has to be in the canonical VirtualFlow format with multiple hierarchical levels as described previously [32], with the only difference in the chemical file format (MOL2 instead of PDBQT). A new parameter *ligand_library_format* was added to the VirtualFlow Ants version of the VFVS control file which specifies the parameters of the virtual screening. The new parameter can have the values “PDBQT” or “MOL2”. Alternatively, the ligands can also be in the PDBQT format, in which case VirtualFlow Ants converts the ligands into the MOL2 format on the fly during the virtual screening. This mode allows to use previously prepared ligand libraries which are in the VirtualFlow PDBQT format, such as the Enamine REAL library containing 1.4 billion compounds which we had previously prepared [32]. The on the fly conversion of the ligands to MOL2 format happens in two steps. In the first step, Open Babel is used to convert the ligand from the PDBQT format to the PDB format, retaining the tautomerization states of the ligands [36,37]. In the second step, SPORES is used with the *completepdb* mode to prepare the MOL2 version of the molecule [38,39]. SPORES prepares ligands and proteins separately, which makes straightforward the input file preparation for virtual screening. During the preparation, it assigns atom and bond types as well as the protonation states using atom and bond information. Additionally, it can generate multiple protonation and tautomeric states, as well as multiple stereoisomers. The complete workflow of the VirtualFlow Ants module can be seen in Figure 2.

The on the fly conversion is very fast (typically a fraction of a second), and therefore does not increase the virtual screening time significantly.

### 3.2. I/O and File Management

Docking using PLANTS generates a relatively large number of output files for each individual molecule, which is virtual screens multiplied by the number of ligands screened. Many of these files can be classified as log files, while others contain the results. As with earlier versions of VirtualFlow, the results files and the log files in VirtualFlow Ants are stored in separate final output folders. All files which contain any of the following regular expressions: .*log, .*constraints.*, .*correspondingNames.*, .*descent.*, .*optimizer.*, .*plantsconfig.*, and .*skippedligands.*. All other files are treated as results files, which includes the docking pose file and the ranking (docking score) files. It is recommended to disable unnecessary output files of PLANTS, such as the writing of protein conformation, in case the protein is rigid and thus identical for all the ligands.

All the processes regarding the on the fly ligand preparation with SPORES and Open Babel, as well as docking with PLANTS, are executed on the local compute nodes and use their memory and local disks if specified in the control file to reduce the I/O for the shared cluster file system.

### 3.3. Configuration and Set Up of VirtualFlow Ants

VirtualFlow Ants is mostly setup in a way similar to how VirtualFlow is setup in general. The main configuration file of VirtualFlow Ants is therefore also the *control file*. Regarding the ligand library format, a new parameter *ligand_library_format* was added to the control file. The new parameter can have the values “PDBQT” or “MOL2”. If the value is “PDBQT”, then the ligands will be converted on the fly from the PDBQT to the MOL2 format as described above.

For the docking scenario which is to be deployed during the virtual screening, one folder has to be created in the input-files folder, and contain a file with the filename config.txt as the main configuration file for the PLANTS docking program. All path names which are specified in this file have to be relative to the tools folder. Protein receptor structures are stored in the folder input-files/receptors.

PLANTS can also be used in a consensus docking procedure within VirtualFlow Ants. This can be done by setting up multiple docking scenarios, which are all carried out for each ligand during the virtual screening. Any number of the supported docking programs and scoring functions of VirtualFlow can take part in the consensus scoring besides PLANTS (e.g., AutoDock Vina [34], QuickVina 2 [35], ADRF [40], QuickVina-W [41], Smina Vinardo [42,43], VinaXB [44], or Vina-Carb [45]), which can be specified in the control file in the section defining the docking scenarios. During the virtual screening, the docking score of each ligand is stored for each docking scenario. After the virtual screening is completed, the consensus score can be obtained by computing the average value of the individual docking scores.

The dedicated web page for VirtualFlow Ants was created (https://virtual-flow.org/virtualflow-ants, accessed 14 May 2021), which describes this new feature to potential users and lists all available additional resources, including documentation.

### 3.4. Test System

As a biomolecular test system for the benchmark studies of VirtualFlow Ants the protein KEAP1 was selected, and as the target site the nuclear factor erythroid 2-related factor 2 (NRF2) binding site. NRF2 orchestrates cellular response to oxidative stress by upregulating detoxifying and antioxidant defense genes [46]. In normal physiological conditions, NRF2 is degraded with the help of KEAP1, an E3 ubiquitin ligase substrate adaptor protein that directly engages NRF2 [47]. The half-life of NRF2 is around 25 min. In response to oxidative, inflammatory, and metabolic stress, key cysteine residues in KEAP1 are covalently modified, preventing KEAP1 from engaging NRF2. This free NRF2 translocates to the nucleus and activates its transcriptional program of approximately 250 genes [48]. Dysregulation of the NRF2 pathway is implicated in a number of diseases, including cancer, diabetes, autoimmune disorders, neurodegenerative disease, metabolic syndrome and diseases of the gastrointestinal tract [49]. Activating NRF2 by inhibiting the protein–protein interaction between KEAP1 and NRF2 is a promising therapeutic strategy pursued by several pharmaceutical companies.

**Figure 3:**
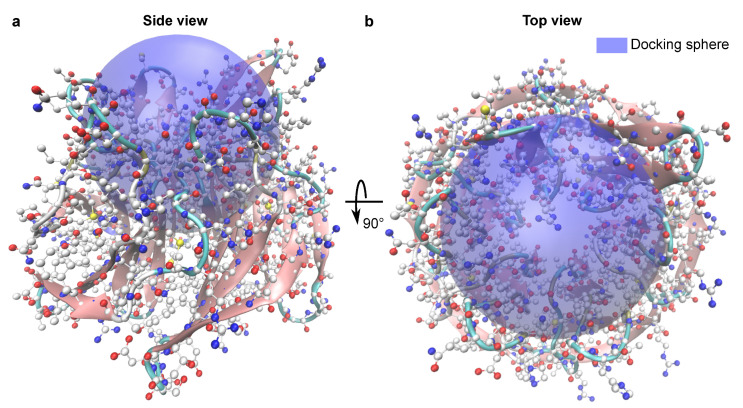
The target protein in the test system, which was chsen to be KEAP1. The target site is the NRF2 protein–protein interaction interface on KEAP1 (PDB ID 5fnq). The side view (**a**) and the top view (**b**) of KEAP1 are shown. The docking sphere of PLANTS which was used in all test runs (blue sphere) was centered at the NRF2 binding interface on KEAP1 and has a diameter of 13 Å.

For KEAP1, multiple structures of high quality are available. The structure that we used has PDB ID 5fnq with the water molecules removed. This structure was selected because it has a relatively high resolution of less than two Angstroms, and no heavy atoms were missing. The structure was prepared with SPORES into the MOL2 file format. The docking sphere used by PLANTS was specified to have a radius of 13 Å, and its center is positioned at coordinates (x,y,z)=(17Å,66Å,31Å) (see Figure 3).

### 3.5. Scaling Behavior

To verify the ability of VirtualFlow Ants to scale to a large number of CPUs, we have tested the scalability in the Google Cloud (https://cloud.google.com/, accessed 14 May 2021). VirtualFlow Ants was run with the KEAP1 as the test system (see above). The docking parameters of PLANTS were set to the default values, except for the speed which was set to *speed4*.

Regarding the ligand library used for this benchmark, we have created a test library of a total of 1.44 billion compounds. This library contains 10 meta-tranches, and each meta-tranche contains 1000 tranches. Each tranche contains 1000 collections, and each collection consists of an identical set of 144 different compounds. Each of the 144 compounds has a molecular weight between 400 and 425 daltons and between three and five rotatable bonds. The ligands were in the PBDQT format, and therefore the newly implemented on the fly conversion from the PDBQT format into the MOL2 format.

**Figure 4:**
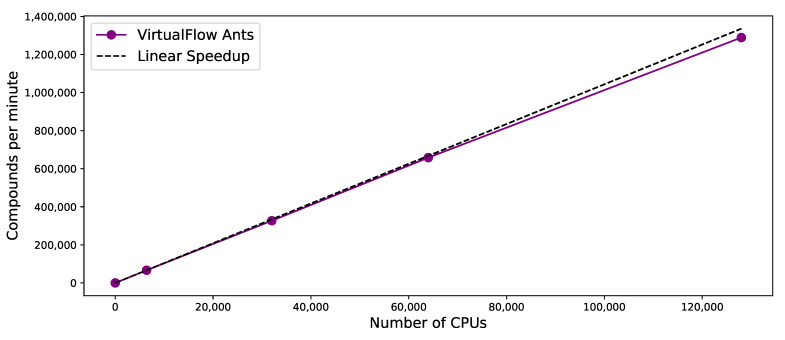
Scaling behavior of VirtualFlow Ants with respect to the number of vCPUs. VirtualFlow Ants was run on the Google Cloud using 6400, 32,000, 64,000 and 128,000 vCPUs. Ligands were in the PDBQT format, and were converted into the MOL2 format required by PLANTS on the fly with Open Babel and SPORES. The number of ligands docked (y-axis) scales approximately linearly with the number vCPUs (purple line). The dashed black line indicates a perfectly linear scaling behavior.

VirtualFlow Ants was run on the Google Cloud using an autoscaling Slurm cluster. The shared cluster filesystem which was deployed was an Elastifile filesystem, which is a high-performance shared network filesystem. The compute nodes of the Slurm cluster were of the type n2d-highcpu-64 (AMD Epyc Rome processors), with external IPs disabled. VirtualFlow was run on 100 compute nodes (using 6400 vCPUs), 500 compute nodes (32,000 vCPUs), 1000 nodes (64,000 vCPUs), and 2000 nodes (128,000 vCPUs). As can be seen in Figure 4, the scaling behavior is virtually linear with respect to the number of vCPUs used.

### 3.6. Parameter Variation

To study the effects of the docking parameters on the virtual screening, we have varied their values and created eight different docking scenarios, which all use the ChemPLP scoring function so that the values can be compared with each other. These eight docking scenarios are listed in Table 1.

**Figure 5:**
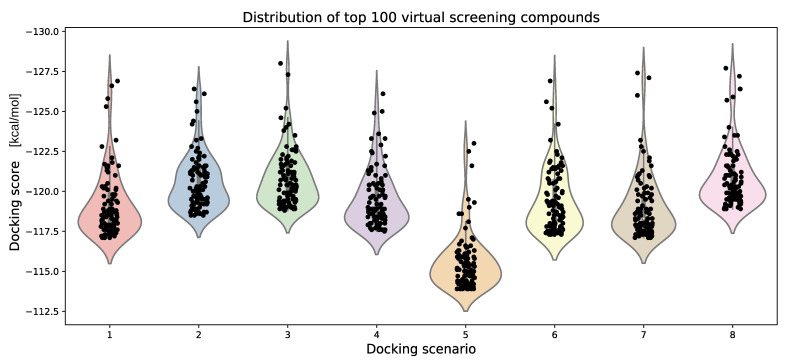
Violin plots of the docking scores of the top 100 ranking compounds for the eight different docking scenarios which use the chemplp scoring function. For each of the eight docking scenarios, approximately 10 million compounds with a molecular weight between 450 and 500 daltons from the Enamine REAL library were screened.

For each of these docking scenarios, we have screened approximately 10 million compounds from the Enamine REAL library with a molecular weight between 450 and 500 daltons. The input library was in the PDBQT format, and therefore the ligands were converted on the fly into the MOL2 format. The distribution of the docking scores of the top 100 compounds can be seen in Figure 5.

**Figure 6:**
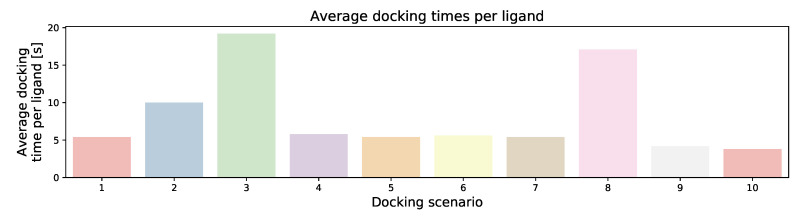
Bar graph showing the average docking time per ligand of the test set, which was used in the above parameter variation benchmark for each of the ten docking scenarios.

As could be expected, with slower speed, the average docking time per ligand increases, and the increase in time is approximately linear relative to the speed parameter (see Figure 6). The accuracy of the docking only marginally decreases with the docking speed, as the average docking scores of the top 100 compounds (AVE100) of docking scenario 1 (speed 4), docking scenario 2 (speed 2) and docking scenario 3 (speed 1) are −118.5, −120.1, and −120.4, respectively, (see Figure 5). The more negative the docking score, the tighter the predicted binding. Reducing the number of ants to 10 seems to slightly improve the results (AVE100 = −118.9). Increasing the number of ants to 50 has reduced the AVE100 value to −115.5. Alternation of the aco_evap parameter to 0.10 and 0.25 (docking scenarios 6 and 7) had minimal effects on the docking scores of the top 100 ranking compounds (AVE100 of −118.75 and −118.3). In docking scenario 8, the aco_sigma value was increased to 1, which slightly increased the docking scores of the top 100 compounds (−120.2) when compared with the default settings (−120.2).

In virtual screenings, an important aspect is the speed of the docking programs, and this depends on the various docking parameters used. We have therefore obtained the average docking times per ligand for each of the ten docking scenarios of Table 1 (see Figure 6). Only docking scenarios 1 to 8 are included, because docking scenarios 9 and 10 use a different scoring function, and thus the docking scores cannot be directly compared with each other.

The fastest two docking scenarios were docking scenarios 9 and 10, in which the plp and plp95 scoring functions were used, respectively. Regarding the docking scenarios 1 to 8 in which the ChemPLP scoring function were used. The docking parameter search_speed, which can have the values 1, 2, and 4, indeed sped the docking times up by a factor approximately represented by the corresponding value.

## 4. Discussion

**Figure 7:**
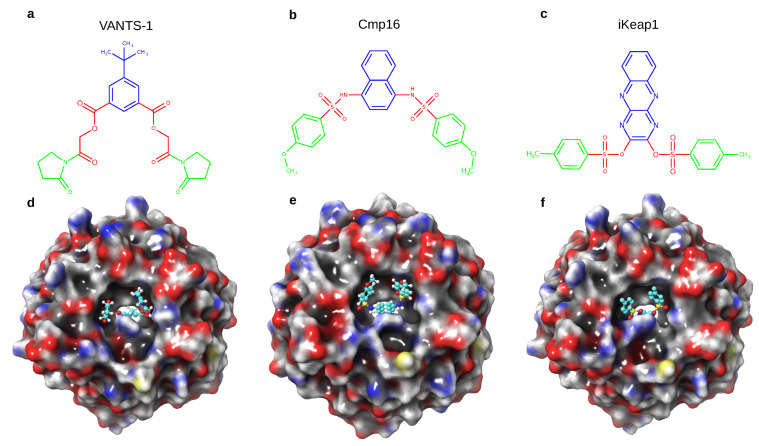
(**a**) Compound VANTS-1 (**b**) the top scoring compound of the 10 million compound screen with docking scenario 1. (**b**,**c**) Compounds Cmp16 and iKeap1, which are similar to compound VANTS-1, and which are experimentally confirmed inhibitors of KEAP1 published previously [32,50]. (**d**) the docking pose of VANTS-1 obtained via PLANTS in docking scenario 1. (**e**,**f**) crystal structure of Cmp16 (PDB ID 4iqk) [50], and docking pose of iKeap1 [32].

In this study, we docked 10 million compounds to the NRF2 binding interface of KEAP1 and compared several docking scenarios where we varied the docking parameters. The top scoring compounds (rank 1) of the benchmark virtual screen involving docking scenario 1 (named VANTS-1) is shown in Figure 7a. This compound has striking similarities with two previously published compounds, Cmp16, experimentally identified by the pharmaceutical company Biogen Idec [50] (Figure 7b), and iKeap1, the inhibitor from our previously published paper [32] (Figure 7c). The predicted binding mode of compound VANTS-1 (Figure 7d) is very similar to the experimentally confirmed binding modes of Cmp16 (Figure 7f) and the docking pose iKeap1 (Figure 7e).

The three molecules, VANTS-1, Cmp16 and iKEAP1, can be compared using their structural motifs, or pharmacophores: All three molecules share an aromatic core structure (depicted in blue in Figure 7), which serves as a mirror plane, along which all molecules are symmetrical. In the case of of iKEAP1, this pharmacophore is comprised of a tricyclic, heteroaromatic pyrazino[2,3-b]quinoxaline, which is simplified to a bicyclic naphthalene in Cmp16. The new molecule VANTS-1 has a further simplification by replacing one aromatic ring with *tert*-butyl as a lipophilic bioisostere. In a symmetrical way, all compounds branch out from the aromatic system using a linker functionality (depicted in red, Figure 7). In the case of iKEAP1, this linker is represented by sulfonates. In the case of Cmp16, sulfonamides are serving the same function, while additionally gaining the ability to provide hydrogen donor functionalities. In VANTS-1, the linker is comprised of 2-oxo-ethyl benzoate linkers, which provide greater flexibility while retaining the hydrogen bond acceptor functionalities found in the original linkers. The last pharmacophore is represented by a cyclic system (depicted in green), Figure 7. Both iKeap1 and Cmp16 harbor aromatic features here in the form of toluene and anisole, respectively. In VANTS-1, this part of the molecule is where the most dramatic change in pharmacophores is found, with a non-aromatic 2-oxopyrrolidine functionality. While having lost its aromaticity compared to the other two molecules, the new moiety in VANTS-1 is still lipophilic. Additionally, the hydrogen acceptor functionality of the anisole moiety in Cmp16 is conserved in the new 2-oxopyrrolidine moiety. The 10 million compounds we screened here are a part of the Enamine REAL library and iKEAP1 belongs to the NCI collection and Cmp16 was from an experimental effort. Hence, we docked iKEAP1 and Cmp16 using PLANTS and we obtained a similar docking score for all the three compounds (VANTS-1 −126.1; Cmp16 −106.4; iKeap1 −107.6). Even though the compound VANTS-1 is similar to Cmp16 and iKeap1, the scaffold is different, and can be highly valuable in drug discovery efforts.

PLANTS will be a great addition to the arsenal of docking programs that VirtualFlow supports. The default setting in PLANTS works well and the speed is conducive for screening ultra-large virtual screening. With the speed setting of 4, one can potentially screen one billion compounds in approximately 15 h day when using 100,000 CPUs. It should be noted that the precise docking time depends on the size of the docking box, the target protein, and other docking parameters. We have noted that the on the fly conversion from PDBQT to MOL2 does not increase the screening time significantly. Since we have already prepared the entire Enamine REAL library in a ready to dock PDBQT format, this on the fly conversion to MOL2 format can be adapted for other docking programs such as GOLD [11,51,52], GLIDE [53,54,55] and LeDOCK [56]. In addition to ultra-large scale screenings PLANTS will be an useful tool for second-stage re-scoring utilizing features like explicit water molecules, protein flexibility and IFP. In challenging cases where a crystal structure is not feasible, PLANTS can be used to include experimental constraints to obtain a docked model of the ligand to the protein, which can be further used to perform virtual medicinal chemistry.

## Figures and Tables

**Table 1:** In this table, the docking scenarios are listed which were included in the parameter variation benchmark studies. The default values which PLANTS uses for the parameters search_speed, aco_ants, and aco_sigma depend on the values of the parameters scoring_function and search_speed. the values highlighted in light blue are the values which were actively set in the configuration file of PLANTS. The remaining values (white background) are the default values which were set by PLANTS. For each of these docking scenarios, approximately 10 million compounds were screened.

Docking Scenario	scoring_Function	Search_Speed	aco_Ants	aco_Eevap	aco_Sigma
1	chemplp	4	default (20)	default (0.15)	default (0.25)
2	chemplp	2	default (20)	default (0.20)	default (0.5)
3	chemplp	1	default (20)	default (0.20)	default (1.25)
4	chemplp	4	10	default (0.15)	default (0.25)
5	chemplp	4	50	default (0.15)	default (0.25)
6	chemplp	4	default (20)	0.10	default (0.25)
7	chemplp	4	default (20)	0.25	default (0.25)
8	chemplp	4	default (20)	default (0.15)	1
9	plp	4	default (20)	default (0.2)	default (0.5)
10	plp95	4	default (20)	default (0.2)	default (1.25)

## Abbreviations

The following abbreviations are used in this manuscript:

MDPI	Multidisciplinary Digital Publishing Institute
DOAJ	Directory of open access journals
TLA	Three letter acronym
LD	Linear dichroism
AVE100	Average docking scores of the top 100 ranking compounds
IFP	Interaction fingerprints
STD	Saturation transfer difference
ACO	Ant colony optimization
KEAP1	Kelch-like ECH-associated protein 1
NRF2	Nuclear factor erythroid 2-related factor 2
